# High-Pressure Injection Injury to the Hand - A Case Report

**DOI:** 10.21980/J8D64W

**Published:** 2022-07-15

**Authors:** Cesar Fortuna, Derek Prince, Daniel Ng, John Costumbrado

**Affiliations:** * Harbor-University of California, Los Angeles, Department of Emergency Medicine, Torrance, CA; ^ University of California, Riverside, School of Medicine, Riverside, CA; † Riverside Community Hospital, Department of Emergency Medicine, Riverside, CA

## Abstract

High-pressure injection injuries to the hand can be surgical emergencies. The injected material and subsequent edema increase the risk for infection, ischemia, compartment syndrome, and limb loss. This is a case report of a 53-year-old male who presented to the emergency department with a superficial two-centimeter laceration to the dorsum of his left hand. He had no obvious signs of infection and the surrounding structures were neurovascularly intact. The patient had been using a gas-powered pressure washer when he sustained the injury to his hand. Physical exam revealed marked crepitus throughout the left hand extending up to the mid forearm. Radiographs of the left hand and left forearm demonstrated extensive subcutaneous emphysema. Hand surgery was emergently consulted and recommended admission for serial exams and antibiotics. This case illustrates a key point: despite initially benign presentations, high-pressure injection injuries should be approached as emergent processes that can be easily overlooked if the proper history and appropriate diagnostic measures are not executed.

**Topics:**

Hand, orthopedics, x-ray, high-pressure injection injury.

**Figure f1-jetem-7-3-v6:**
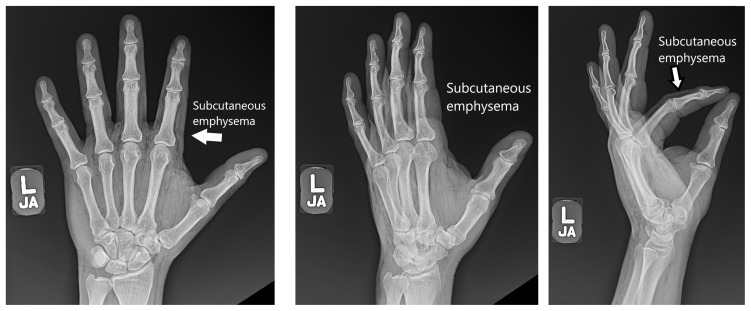


**Figure f2-jetem-7-3-v6:**
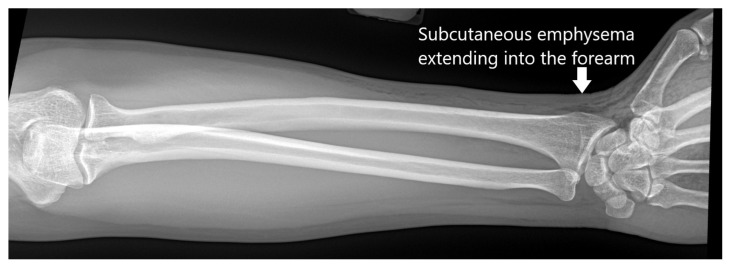


**Figure f3-jetem-7-3-v6:**
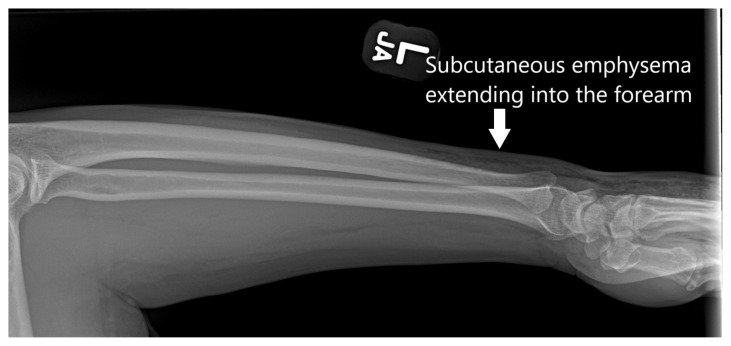


## Brief introduction

[Fig f1-jetem-7-3-v6][Fig f2-jetem-7-3-v6][Fig f3-jetem-7-3-v6]High-pressure injection injuries of the hand are potentially surgical emergencies. Injuries may often appear deceivingly mild in nature and on superficial evaluation, can often be mistaken for a simple abrasion, puncture, or laceration.^1^ Given the high-pressure nature of these injuries, deeper structures are often involved, leading to infection, ischemia, and compartment syndrome.^2^ Failure to recognize these injuries can lead to irreversible damage and loss of limb.^3^ These injuries can have devastating consequences, and the impact of the trauma is often compounded given that high-pressure injection injuries most commonly affect males who work with their hands for a living.^1^

## Presenting concerns and clinical findings

A 53-year-old, right-hand dominant male with no significant past medical history presented to the emergency department with a laceration to the dorsum of his left hand. He sustained the injury using a gas-powered pressure washer. At the time of the injury, the power washer contained only water and not detergent or other soaps. On exam, the patient had a linear two-centimeter laceration, which appeared superficial in nature without exposure of tendon or bone. Motor and sensory functions were intact, and the hand was well-perfused on exam. He had marked crepitus palpable throughout his hand and extending up to the mid-forearm.

## Significant findings

Plain radiographs of the left hand and forearm demonstrated extensive subcutaneous emphysema. The air can be seen as lucent striations tracking along the second and third fingers as well as along the dorsum of the hand and wrist. There is also diffuse soft tissue emphysema surrounding the metacarpophalangeal joints. Lab analysis did not show any significant acute abnormalities.

## Patient course

Hand surgery was immediately consulted, and the patient was admitted for serial examinations as well as intravenous antibiotics. Hand surgery suspected that his injury would be unlikely to develop serious sequelae given that the injected substance was only water and not a more harmful substance such as paint or oil. The patient did well throughout his hospital course and did not require surgical intervention. He was discharged home with oral antibiotics and did not develop any complications.

## Discussion

High-pressure injection injuries are uncommon injuries that typically occur in the nondominant hand of young-to-middle aged males, in particular laborers.^4^–^5^ These injuries account for 1 out of 600 hand traumas, with 1–4 cases seen at the average trauma center per year.^1^ In a retrospective review of 20 cases involving high-pressure injection injuries, the incidence over a 10-year time period was 2.1 cases per year, with the nondominant hand injured in most cases (63%) and the index finger being the most common site of injury (55% of reported incidents).^4^ These injuries appear with small superficial wounds, minimal pain, and little loss of function which can lead to underestimating the degree of damage.^2^ This can result in a costly delay of appropriate treatment and can ultimately lead to severe morbidity or loss of limb since the rate of amputation following these injuries is 30%.^5^ Accordingly, hand injuries caused by high-pressure injection should be considered surgical emergencies.

These injuries often result from the incorrect utilization of equipment that create very high ejection pressures.^6^ Ejection pressures must be at least 100 pounds per square inch (psi) to penetrate human skin. Most high-pressure guns and injectors reach pressures of up to 2,000 psi.^7^,^8^ The immense pressure released from the injector tip alone is sufficient to cause considerable mechanical damage to soft tissue such as muscle and neurovascular structures. The injection of toxic substances such as paints and solvents further compounds the risk of severe complications such as limb-threatening ischemia and compartment syndrome.

Despite the serious nature of these injuries, patients arriving to the emergency department immediately after sustaining a high-pressure injection injury classically present with small puncture wounds to the hand with little external evidence of the full extent of the injury. It is critical that providers caring for this type of injury are aware of the proper evaluation and management. A study done in 2008 reported 3 of their 8 patients presented immediately after an injection injury with a mild condition that deteriorated rapidly in the following 6 hours.^6^ Surgical debridement within 6 hours of injury is often necessary in these cases to reduce the risk of amputation.^5^

The type of injected material is one of the main contributing factors to adverse outcomes. The most commonly injected substances are fuel, oil, grease, paint solvent, air, water, and cement, with paint being the most commonly injected material.^9^–^11^ Air and water have the best prognosis in injection injuries because they lead to only a minimal inflammatory response and are absorbed over time. Oil-based paints have been reported to be more inflammatory and have higher complication rates when injected in comparison to water-based paints.^9^ Organic solvents, such as turpentine and paint thinners, are extremely cytotoxic and cause liquefactive necrosis of tissues leading to the worst prognosis of these materials.^10^ Additionally, infection is a concern because the injection of a foreign substance into tissue can also introduce bacteria. Rates of infection following injection injuries vary widely in the literature from 1.6% to 60% with most being polymicrobial.^1^

It is critical to obtain a thorough history from the patient to determine the nature of the injury. Practitioners should also perform a dedicated physical exam of the affected hand, forearm, elbow, upper arm, and axilla to assess the extent of the injury and to evaluate for signs of neurovascular compromise. In terms of labs, some degree of leukocytosis can be expected within the first few hours after injury, but the white blood cell count can be useful to monitor for signs of secondary infection.^12^ For imaging, plain radiographs assist in the visualization of radiopaque injected substances and in detecting subcutaneous emphysema. Computed tomography (CT) and magnetic resonance imaging (MRI) scans are not typically necessary but can be helpful in assessing the extent of soft tissue damage. Extensive review of the literature revealed no specific information on sensitivity or specificity for imaging high-pressure injection injuries.

Treatment of high-pressure injection injuries in which organic solvents, paints, and fuel-based substances were injected may require surgical debridement to control the inflammatory response and to ensure the compartments of the affected limb are properly decompressed if signs of compartment syndrome are evident. Early surgical management, when necessary, is critical to reduce the risk of long-term morbidity in these patients.^12^ Injection injuries with air or water can typically be treated with a non-surgical approach including close observation if no signs of infection or compartment syndrome are present. In addition, administering a tetanus booster (if required) and a third-generation cephalosporin for prophylactic coverage of both gram-negative and gram-positive bacteria can improve patient outcomes.^9^

This patient’s initial presentation seemed benign with a 2 cm superficial laceration to the left hand, but key history and physical exam findings were imperative in revealing a high-risk mechanism of injury and marked crepitus to the hand extending to the forearm. Plain radiographs confirmed subcutaneous emphysema and an appropriate emergent consult to hand surgery was conducted. Fortunately, due to the more benign nature of the injection material, this patient did not require surgical intervention and was treated initially with intravenous antibiotics and then discharged on oral antibiotics without complications. Immediate recognition of the mechanism of injury, an appropriate physical exam, and obtaining plain radiographs are vital steps to quickly assess the extent of high-pressure injection injuries and decrease overall morbidity and complications.

## Supplementary Information




















