# Supplementary figures and images for: High-Pressure Injection Injury to the Hand - A Case Report
Source: J Educ Teach Emerg Med. 2022 Jul 15;7(3):V6–9. doi: 10.21980/J8D64W (PMC10332702; doi:10.21980/J8D64W)

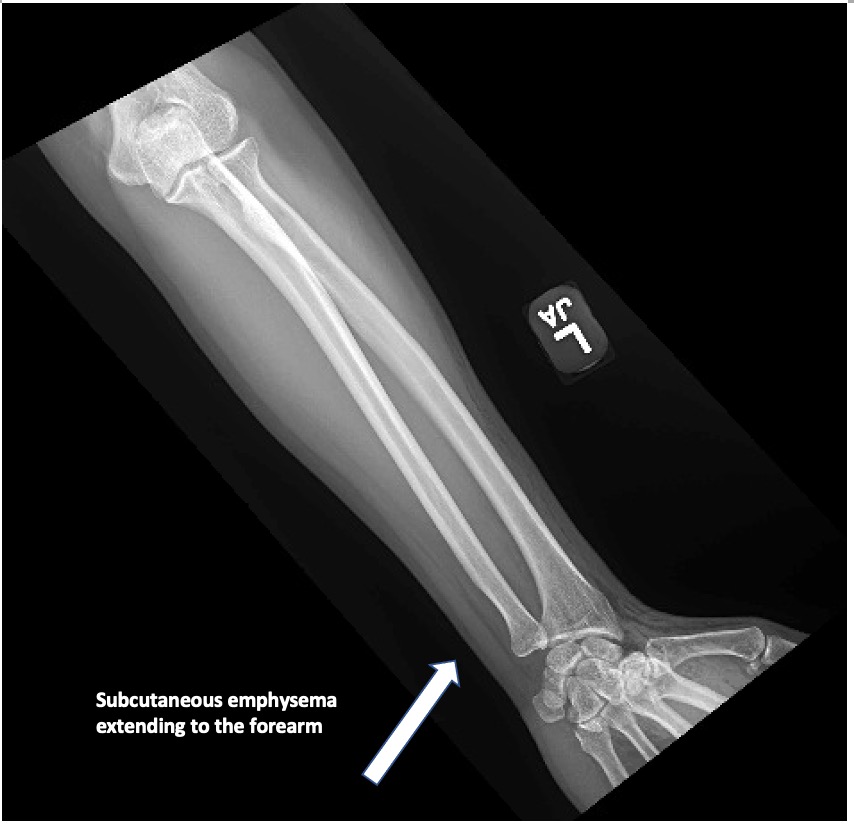

Supplement: Supplementary file 1 [file jetem-7-3-v6-supp1.jpeg]

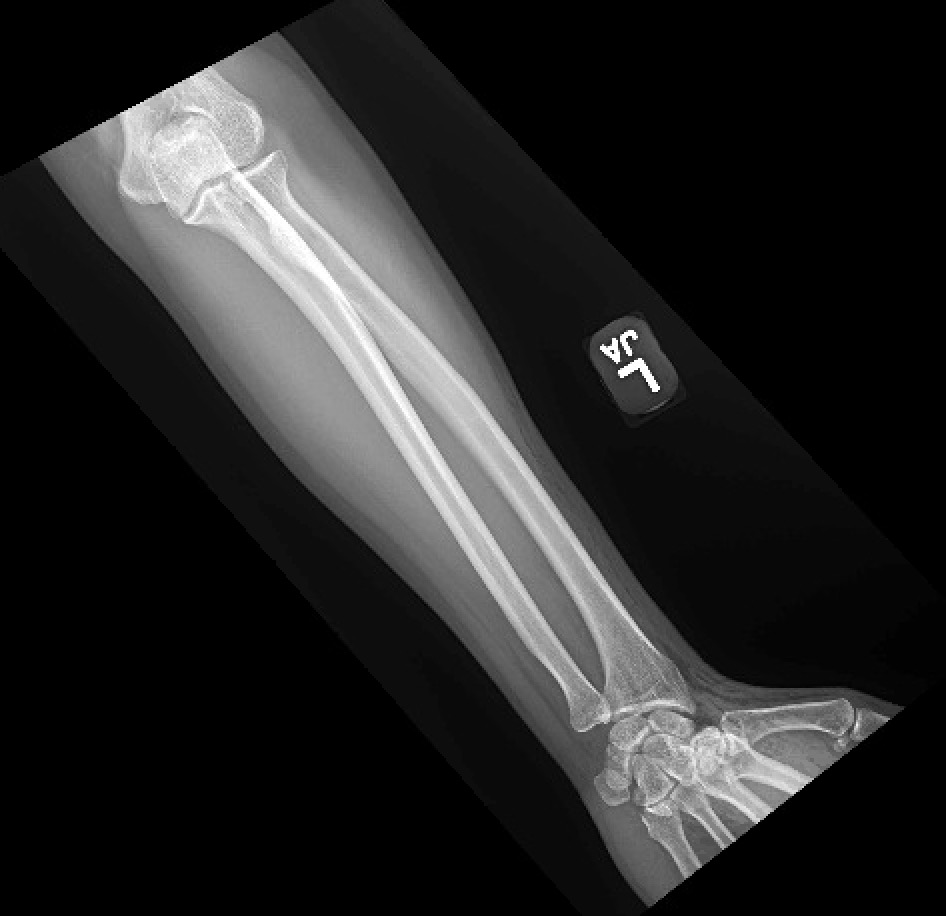

Supplement: Supplementary file 2 [file jetem-7-3-v6-supp2.jpeg]

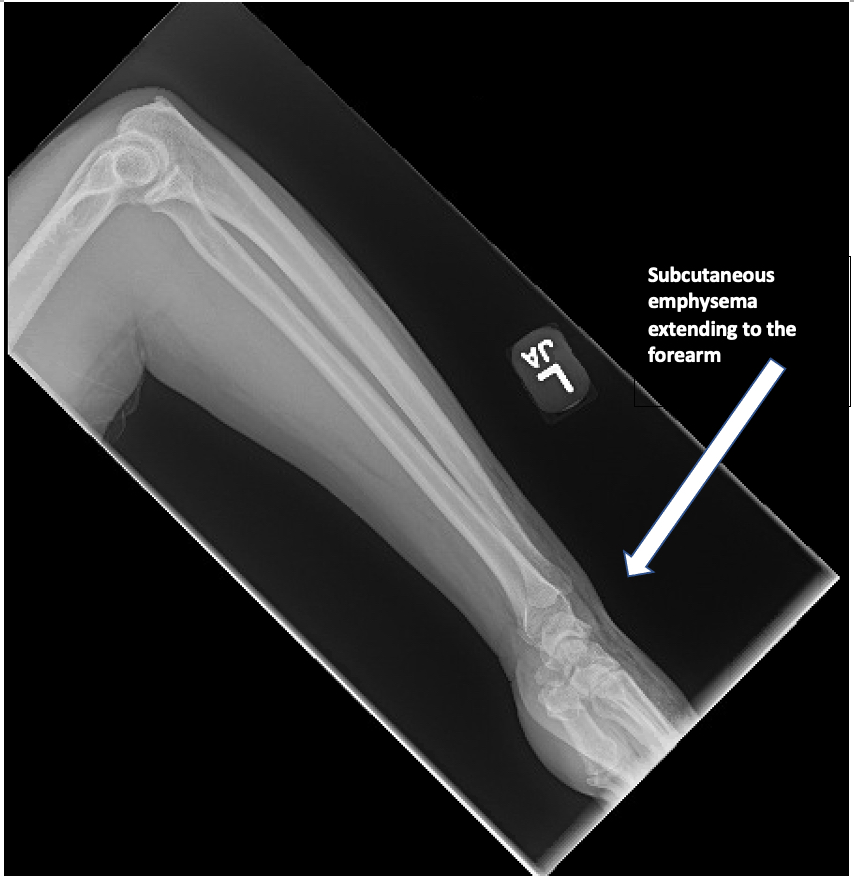

Supplement: Supplementary file 3 [file jetem-7-3-v6-supp3.jpg]

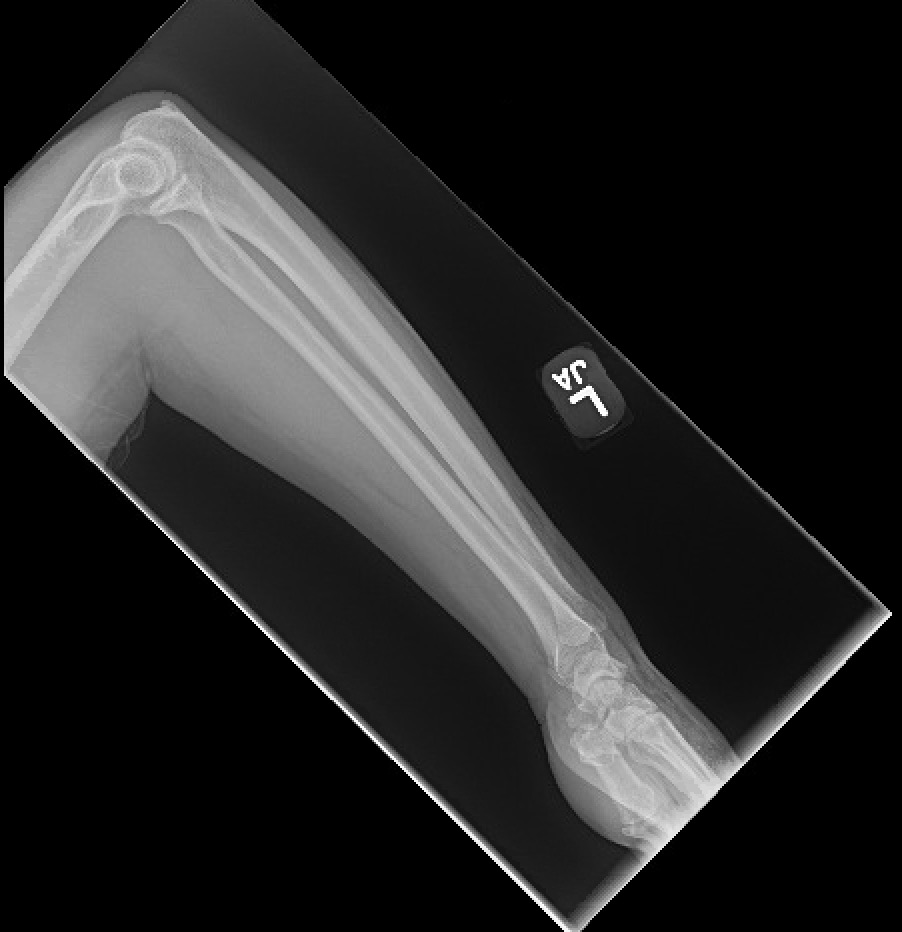

Supplement: Supplementary file 4 [file jetem-7-3-v6-supp4.jpeg]

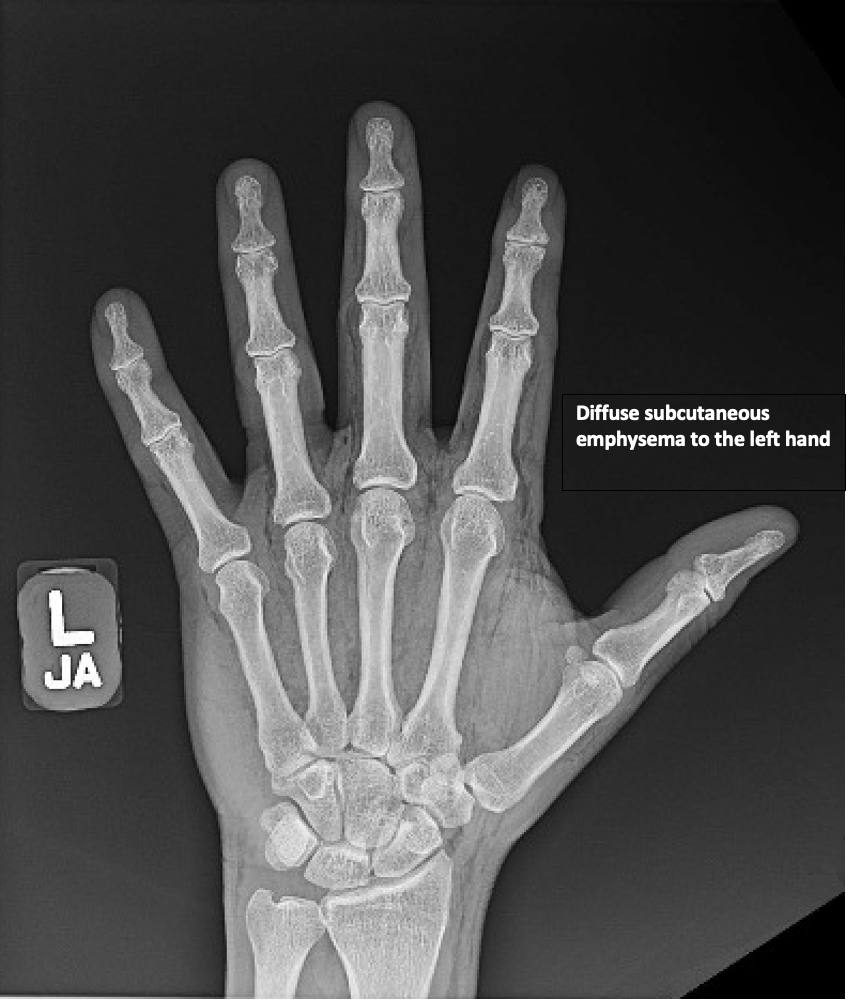

Supplement: Supplementary file 5 [file jetem-7-3-v6-supp5.jpeg]

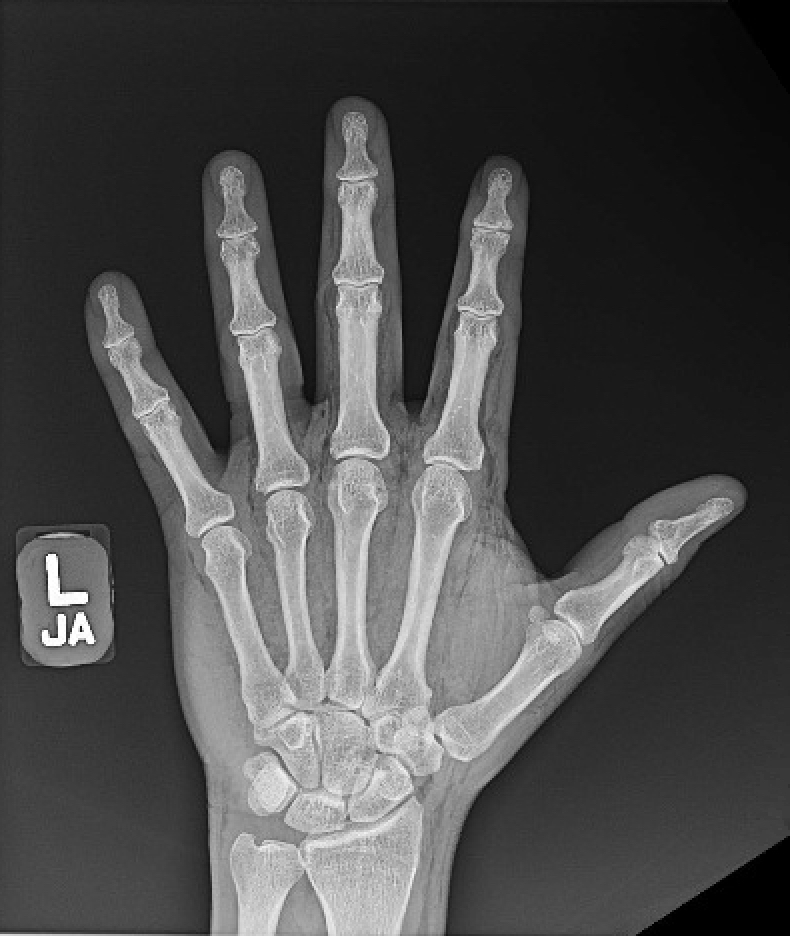

Supplement: Supplementary file 6 [file jetem-7-3-v6-supp6.jpeg]

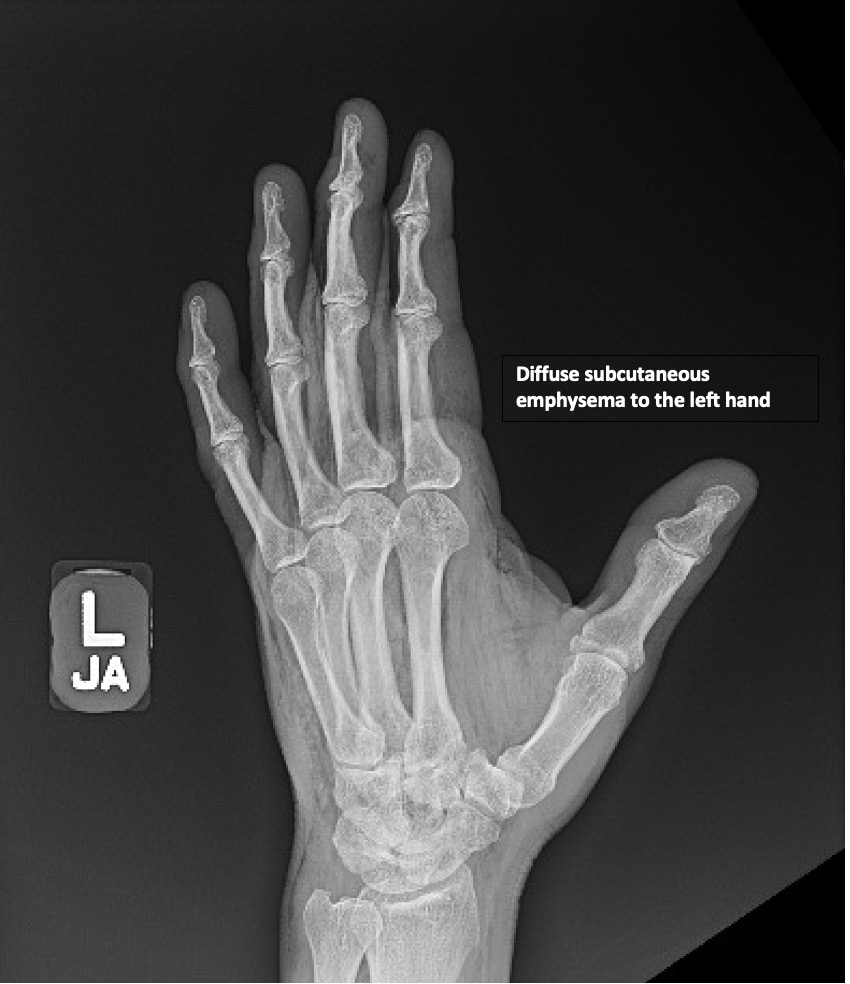

Supplement: Supplementary file 7 [file jetem-7-3-v6-supp7.jpeg]

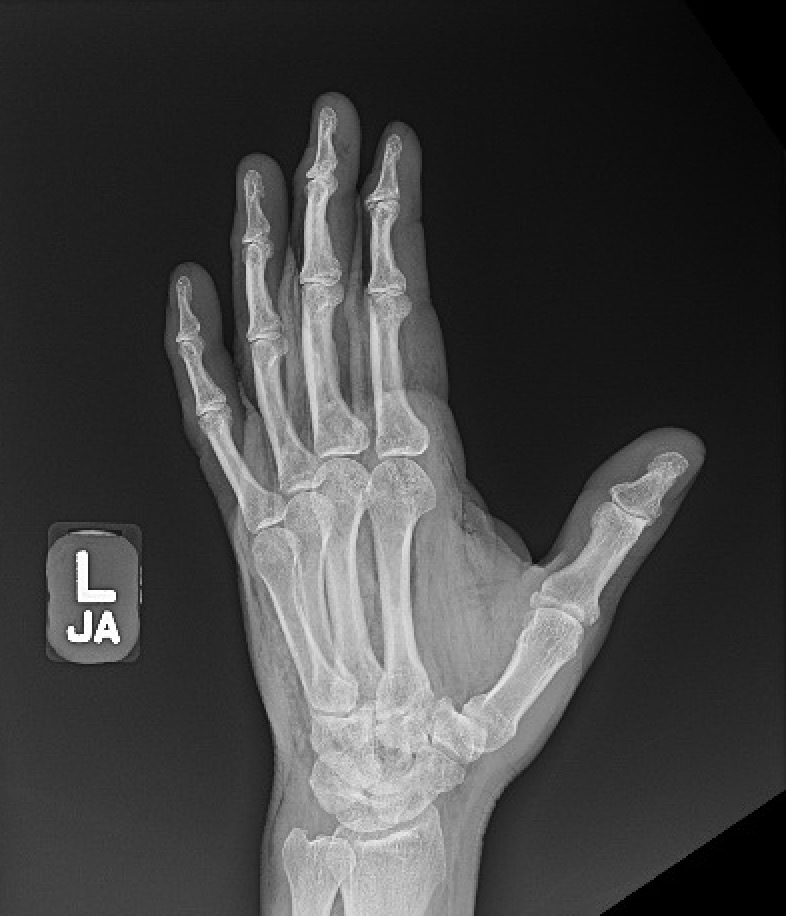

Supplement: Supplementary file 8 [file jetem-7-3-v6-supp8.jpeg]

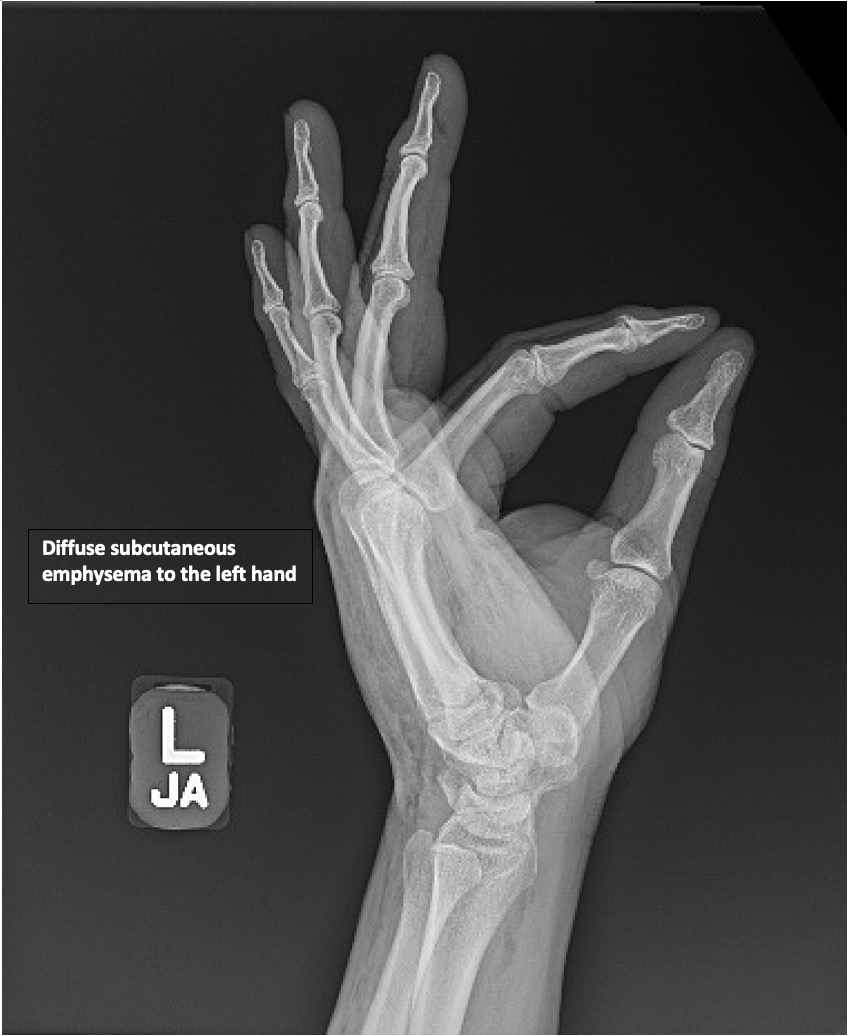

Supplement: Supplementary file 9 [file jetem-7-3-v6-supp9.jpeg]

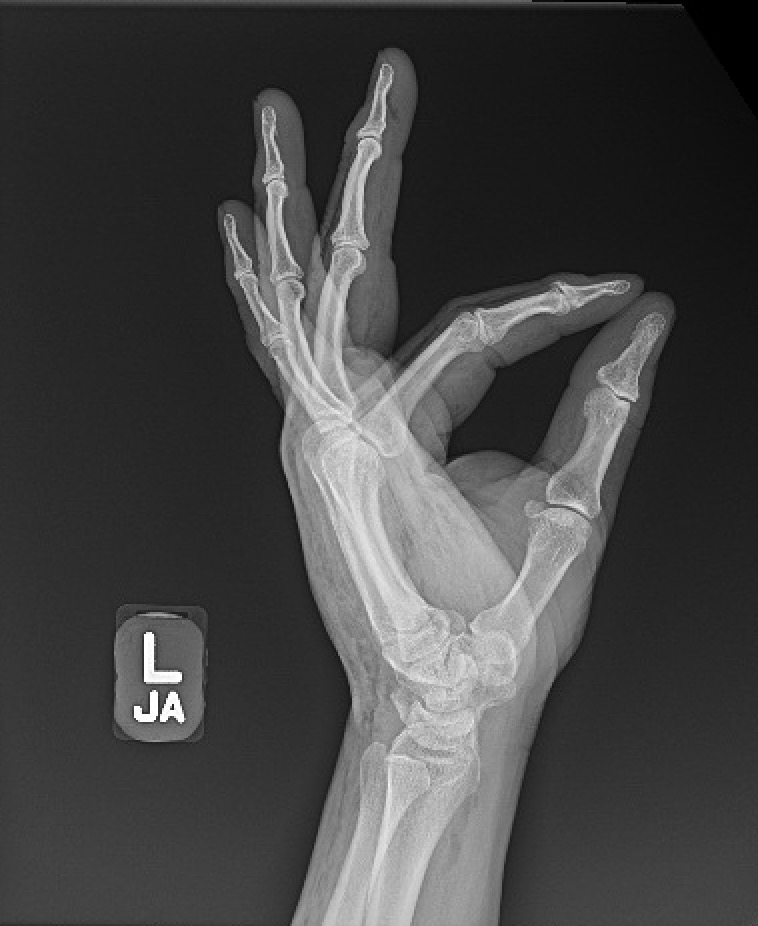

Supplement: Supplementary file 10 [file jetem-7-3-v6-supp10.jpeg]
